# Clinical Applications of Gamma Delta T Cells with Multivalent Immunity

**DOI:** 10.3389/fimmu.2014.00636

**Published:** 2014-12-11

**Authors:** Drew C. Deniger, Judy S. Moyes, Laurence J. N. Cooper

**Affiliations:** ^1^Surgery Branch, National Cancer Institute, Bethesda, MD, USA; ^2^Division of Pediatrics, University of Texas MD Anderson Cancer Center, Houston, TX, USA; ^3^The University of Texas Graduate School of Biomedical Sciences, UT MD Anderson Cancer Center, Houston, TX, USA

**Keywords:** cancer, immunotherapy, γδ T cells, adoptive T-cell therapy, T-cell receptor, allogeneic transplantation, chimeric antigen receptors, artificial APC

## Abstract

γδ T cells hold promise for adoptive immunotherapy because of their reactivity to bacteria, viruses, and tumors. However, these cells represent a small fraction (1–5%) of the peripheral T-cell pool and require activation and propagation to achieve clinical benefit. Aminobisphosphonates specifically expand the Vγ9Vδ2 subset of γδ T cells and have been used in clinical trials of cancer where objective responses were detected. The Vγ9Vδ2 T cell receptor (TCR) heterodimer binds multiple ligands and results in a multivalent attack by a monoclonal T cell population. Alternatively, populations of γδ T cells with oligoclonal or polyclonal TCR repertoire could be infused for broad-range specificity. However, this goal has been restricted by a lack of applicable expansion protocols for non-Vγ9Vδ2 cells. Recent advances using immobilized antigens, agonistic monoclonal antibodies (mAbs), tumor-derived artificial antigen presenting cells (aAPC), or combinations of activating mAbs and aAPC have been successful in expanding gamma delta T cells with oligoclonal or polyclonal TCR repertoires. Immobilized major histocompatibility complex Class-I chain-related A was a stimulus for γδ T cells expressing TCRδ1 isotypes, and plate-bound activating antibodies have expanded Vδ1 and Vδ2 cells *ex vivo*. Clinically sufficient quantities of TCRδ1, TCRδ2, and TCRδ1^neg^TCRδ2^neg^ have been produced following co-culture on aAPC, and these subsets displayed differences in memory phenotype and reactivity to tumors *in vitro* and *in vivo*. Gamma delta T cells are also amenable to genetic modification as evidenced by introduction of αβ TCRs, chimeric antigen receptors, and drug-resistance genes. This represents a promising future for the clinical application of oligoclonal or polyclonal γδ T cells in autologous and allogeneic settings that builds on current trials testing the safety and efficacy of Vγ9Vδ2 T cells.

## Introduction

γδ T cells possess a combination of innate and adaptive immune cell qualities rendering them attractive for immunotherapy (1–3). They can produce inflammatory cytokines, directly lyse infected or malignant cells, and establish a memory response to attack pathogens upon re-exposure. γδ T cells are defined by expression of γ and δ heterodimer of T cell receptor (TCR) chains (TCRγ/TCRδ) that directs intracellular signaling through associated CD3 complexes (4). The γδ T-cell lineage (1–5% of circulating T cells) can be contrasted to the more prevalent αβ T cell lineage (~90%) in peripheral blood, which expresses TCRα/TCRβ heterodimers and also signals through associated CD3 complexes (5, 6). CD4 and CD8 co-receptors on αβ T cells assist binding of TCRαβ chains to the major histocompatibility complex (MHC) presenting processed peptides (7–9). In contrast, TCRγδ directly binds to an antigen’s superstructure independent of the MHC/peptide complexes and, as a result, CD4 and CD8 are uncommon on γδ T cells (10, 11). Given that antigen recognition is achieved outside of MHC/peptide-restriction, γδ T cells have predictable immune effector functions mediated through their TCR and have potential use as universal (“off-the-shelf”) allogeneic T-cell therapies (12).

Functional responses by γδ T cells can be stratified by the variable (V) region of the TCRδ chain. In humans, the TCRδ locus (*TRD*) lies within the TCRα locus (*TRA*). Three unique Vδ alleles, *TRDV1*, *TRDV2*, and *TRDV3*, code for TCRδ1, TCRδ2, and TCRδ3, respectively. Additionally, shared Vδ and Vα variable regions exist in *TRDV4*/*TRAV14*, *TRDV5*/*TRAV29*, *TRDV6*/*TRAV23*, *TRDV7*/*TRAV36*, and *TRDV8*/*TRAV38-2* loci. Recombination of these shared V alleles with a *TRA* junction region (*TRAJ*) results in TCRα14, TCRα29, TCRα23, TCRα36, and TCRα38-2, respectively, but recombination of these shared V alleles with *TRD* junction (*TRDJ*) and diversity (*TRDD*) regions results in TCRδ4, TCRδ5, TCRδ6, TCRδ7, and TCRδ8, respectively (13). Expression of TCRγδ heterodimers on the T-cell surface in the thymus inhibits recombination of TCRβ-chain locus during the CD4^neg^CD8^neg^ stage thereby committing the T cell to the γδ T-cell lineage (14). This double negative status is typically maintained upon exit from the thymus, most likely because co-receptors are dispensable for functional TCRγδ binding to antigens (15). However, the thymus is not required to complete all γδ T-cell development, as many γδ T cells directly take up residence in peripheral tissues following exit from the bone marrow and exhibit immediate effector functions against pathogens (16). Thymus-independent “resident” γδ T cells can be found in the mucosa, tongue, vagina, intestine, lung, liver, and skin and can comprise up to 50% of the T-cell populations in intestinal epithelial lymphocytes (17, 18). In contrast, circulating γδ T cells can be found in the blood and lymphoid organs, and are dominated by γδ T cells preferentially expressing TCRδ2 isotype (commonly referred to as Vδ2 cells). Indeed, γδ T cells expressing the TCRδ1 isotype (commonly referred to as Vδ1 cells) are frequently found within tissues (19, 20). Vδ2 cells have preferred pairing with TCRγ9 (Vγ9Vδ2 cells), but broad γ-chain pairing is observed in Vδ1 cells and Vδ1^neg^Vδ2^neg^ cells, a generic grouping of all other non-Vδ1/Vδ2 T cells (12, 19). Therefore, γδ T cells are distributed across an array of anatomical locations with a range of TCRγδ variable region expression.

Human TCRγδ ligands are MHC/peptide complex-independent and are therefore conserved amongst unrelated individuals. Most of the known human ligands are specific for TCRδ1 or TCRδ2. TCRγ1/TCRδ1 (alternatively termed Vγ1Vδ1) heterodimers have specificity for MHC Class-I chain-related A (MICA) (21, 22), a molecule participating in evasion of immune surveillance following viral infection and expressed on tumor cells as it is involved in the cellular stress response (23). MICA is also one of the ligands for NKG2D, which is expressed on γδ T cells, αβ T cells, and natural killer (NK) cells (23, 24). Both Vγ1Vδ1 and Vγ2Vδ1 recognize non-polymorphic MHC molecule CD1c (25), and Vγ5Vδ1 is a receptor for α-galactosylceramide-CD1d complexes commonly described in the activation of natural killer T (NKT) cells which, like γδ T cells, have both innate and adaptive immune functions and recognize conserved ligands amongst unrelated individuals (26, 27). γδ T cells can have specificity for virus as cytomegalovirus (CMV)-reactive Vγ8Vδ1 cells have been isolated from umbilical cord blood from infected newborns (28). Vδ1 cells have also been associated with immunity to human immunodeficiency virus (HIV), but the precise HIV ligands for TCRδ1 have not been determined (29). Bacterial alkylamines and *Listeria monocytogenes* are recognized by Vδ2 cells when paired with Vγ2 (30–32). Vγ9Vδ2 cells are the most extensively studied sub-group of human γδ T cells and their ligands include phosphoantigens [isopentenyl pyrophosphate (IPP)], F_1_-ATPase expressed on the cell surface, apolipoprotein A-I, and *Mycobacterium tuberculosis* (33–37). Moreover, Vγ9Vδ2 cells controlled and prevented lethal Epstein–Barr virus (EBV)-transformed leukemia xenografts in immunocompromised mice (4), and *in vitro* and *in vivo* data suggested that Vδ1 cells are also specific for EBV (38, 39). In contrast to Vδ1 and Vδ2 cells, very little is known about human γδ T cells expressing other TCRγδ alleles except for indirect evidence of Vδ3 cell’s immunity against CMV and HIV (40, 41). Given the multivalent nature of γδ T cells, harnessing γδ T cells populations with polyclonal TCR repertoire is attractive for adoptive immunotherapy.

## γδ T-Cell Clinical Experience

**Figure 1:**
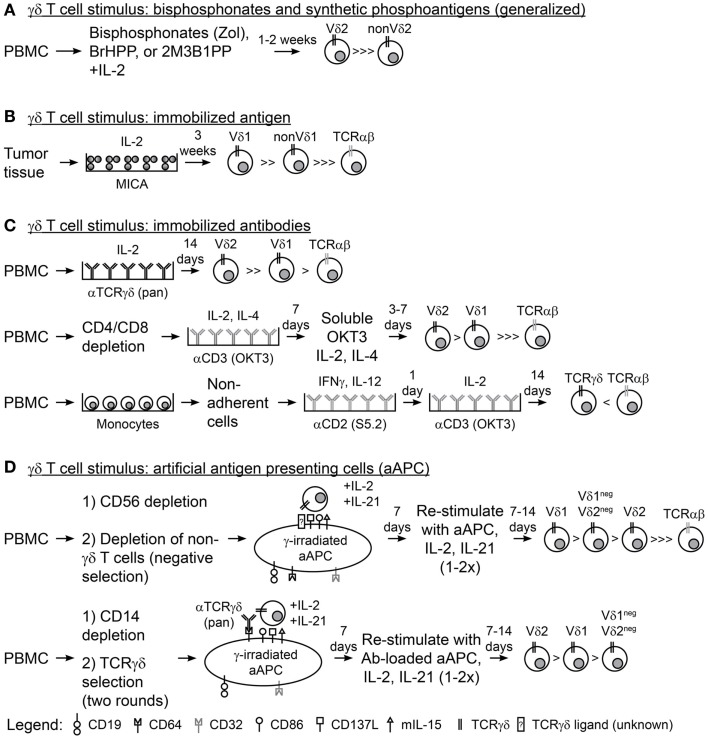
**Methodologies for expanding γδ T cells *ex vivo***. **(A)** A generalized schematic for the use of aminobisphosphonates (Zol, zoledronic acid) or synthetic phosphoantigens (BrHPP, bromohydrin pyrophosphate; 2M3B1PP, 2-methyl-3-butenyl-1-pyrophosphate) and interleukin-2 (IL-2) to expand γδ T cells from peripheral blood mononuclear cells (PBMC). **(B)** Plate-bound MHC class-I chain-related (MICA) and IL-2 were used to expand γδ T cells from colon and ovarian tumor tissues. **(C)** Immobilized antibodies (Ab) were used to expand γδ T cells from PBMC in three scenarios: (top) PBMC directly stimulated with anti-pan-TCRγδ Ab and IL-2, (middle) PBMC depleted of CD4 and CD8 T cells followed by two rounds of stimulus with anti-CD3 Ab (OKT3), IL-2, and IL-4, and (bottom) PBMC were depleted of non-adherent cells, stimulated with anti-CD2 Ab (S5.2), interferon-γ (IFNγ), and IL-12, then stimulated with OKT3 and IL-2. **(D)** Schematic for the use of artificial antigen presenting cells (aAPC) to expand γδ T cells from PBMC in two scenarios: (top) PBMC was depleted of CD56^+^ NK cells then of other non-γδ T cells (TCRγ/δ+ magnetic bead kit) so that γδ T cell were isolated by “negative selection” and co-cultured recursively with aAPC, IL-2, and IL-21 for 2–3 rounds of stimulation; (bottom) PBMC was depleted of CD14^+^ monocytes and “positively selected” with TCRγδ magnetic beads then co-cultured recursively with anti-TCRγδ Ab-loaded aAPC, IL-2, and IL-21 for 2–3 rounds of stimulation.

Immunotherapy with γδ T cells requires their activation and expansion as they comprise only a small percentage of circulating T cells. Interleukin-2 (IL-2) and activating CD3 antibody (OKT3), commonly used for the propagation of αβ T cells directly from peripheral blood mononuclear cells (PBMC), do not reliably expand γδ T cells without further manipulation and so alternative approaches are needed. Aminobisphosphonates, e.g., Zoledronic Acid (Zol), used in the treatment of bone-related diseases, e.g., osteoporosis, resulted in *in vivo* propagation of γδ T cells, and the use of aminobisphosphonates has been subsequently translated into laboratory practice to grow γδ T cells *ex vivo* (Figure 1A) (42, 43). Aminobisphosphonates inhibit cholesterol synthesis and result in the accumulation of phosphoantigen intermediates in the mevalonate–CoA pathway, including IPP, a ligand for Vγ9Vδ2 (44). However, only the Vγ9Vδ2 T-cell subset is reactive to cells treated with phosphoantigens (45, 46). Synthetic phosphoantigens, e.g., bromohydrin pyrophosphate (BrHPP) (47) and 2-methyl-3-butenyl-1-pyrophosphate (2M3B1PP) (48), can mimic aminobisphosphonates and stimulate Vγ9Vδ2 T cells for proliferation.

These reagents have been transitioned to the clinic for investigational treatments of cancer and HIV (Table 1) (49, 50). Six trials have evaluated the ability of aminobisphosphonates or BrHPP to generate *in vivo* expansions of Vγ9Vδ2 T cells to fight leukemia/lymphoma (51, 52), melanoma (52), renal cell carcinoma (RCC) (52, 53), hormone-refractory prostate cancer (HRPC) (54), breast cancer (55), and HIV (56). These trials established safety of large Vγ9Vδ2 T cell expansions *in vivo* and generated a total of nine objective responses (11.3%; *N* = 80) but no complete responses (CR) as anti-tumor therapies. Six clinical trials have used either Zol, BrHPP, or 2M3B1PP to expand autologous Vγ9Vδ2 T cells *ex vivo* and these cells were directly infused (three trials with added IL-2 infusion and three without) for treatment of RCC (57–59), non-small cell lung cancer (NSCLC) (60, 61), and colorectal cancer (CRC) (62). Direct infusion of Vγ9Vδ2 T cells was established as a safe regimen and a total of eight objective responses (11.3%; *N* = 71) were detected, including one CR (1.4%; *N* = 71) (62). Three trials have evaluated the combination of adoptive transfer of *ex vivo* expanded Vγ9Vδ2 T cells followed by Zol administration to boost their *in vivo* proliferation. Multiple myeloma (63), RCC (64), and multiple metastatic tumors (melanoma, CRC, gastrointestinal tumors, ovarian cancer, breast cancer, cervical cancer, and bone cancer) (65) were treated with this combination, which was established to be safe, and four objective responses (13.8%; *N* = 29) were observed, two of which were CRs (6.9%; *N* = 29) treating intermediate-stage RCC (64) and breast cancer (65). Thus, adoptive transfer and *in vivo* expansions of Vγ9Vδ2 T cells are safe therapeutic modalities and can result in objective clinical responses in the treatment of cancer.

**Table 1:** **Clinical responses from γδ T cells**.

Year	Treatment	Disease (*N*)	Total (*N*)	OR (%)	CR (%)	Reference
1996	Allogeneic HSCT depleted of αβ T cells with TBI	ALL	74	43/74 (58%)	25/43 (58%)	(68)
		AML	
		CLL	
2003	Pamidronate and IL-2	MM (8)	19	3/19 (16%)	0/19 (0%)	(51)
		FCL (4)	
		CLL (4)	
		MZL (2)	
		IC (1)	
2007	Zol vs. Zol and IL-2	HRPC (18)	18	3/18 (17%)	0/18 (0%)	(54)
2007	2M3B1PP-expanded autologous Vδ2 T cells and IL-2	RCC (7)	7	3/7 (43%)	0/7 (0%)	(57)
2007	Allogeneic HSCT depleted of αβ T cells	ALL (77)	153	100/153 (65%)	36/153 (24%)	(66)
		AML (76)	
2008	BrHPP-expanded Vδ2 T cells and IL-2	RCC (10)	10	0/10 (0%)	0/10 (0%)	(58)
2009	Zol and IL-2	HIV (10)	10	N/D	N/D	(56)
2009	Zol-expanded Vγ9Vδ2 T cells, Zol, and IL-2	MM (6)	6	0/6 (0%)	0/6 (0%)	(63)
2010	Zol-expanded Vγ9Vδ2 T cells	NSCLC (10)	10	0/10 (0%)	0/10 (0%)	(60)
2010	Zol and IL-2	Breast cancer (10)	10	1/10 (10%)	0/10 (0%)	(55)
2010	BrHPP-expanded Vδ2 T cells and IL-2	RCC (18)	28	0/28 (0%)	0/28 (0%)	(59)
		GI-cancer (4)	
		CRC (3)	
		Breast cancer (2)	
		EOC (1)	
2011	Zol-expanded Vγ9Vδ2 T cells	NSCLC (15)	15	0/10 (0%)	0/10 (0%)	(61)
2011	BrHPP-expanded Vδ2 T cells, Zol, and IL-2	RCC (11)	11	1/11 (9%)	1/11 (9%)	(64)
2011	Zol and IL-2	RCC (12)	12	0/12 (0%)	0/12 (0%)	(53)
2011	Zol-expanded Vγ9Vδ2 T cells and Zol	Melanoma (7)	18	3/12 (25%)	1/12 (8%)	(65)
		CRC (3)	
		GI-cancer (2)	
		EOC (2)	
		Breast cancer (2)	
		Cervical cancer (1)	
		Bone cancer (1)	
2012	Zol and IL-2	RCC (7)	21	2/21 (10%)	0/21 (0%)	(52)
		Melanoma (6)	
		AML (8)	
2013	Zol-expanded Vγ9Vδ2 T cells	CRC (6)	6	5/6 (83%)	1/6 (17%)	(62)
2014	CD4/CD8-depleted haploidentical PBMC, Zol, and IL-2	T-NHL (1)	4	3/4 (75%)	3/4 (75%)	(71)
		AML (1)	
		SPL (1)	
		MM (1)	

*A survey was taken of clinical trials that reported the use of aminobisphosphonates, synthetic phosphoantigens, direct infusion of *ex vivo* expanded γδ T cells, combinations of aminobisphosphonates/synthetic phosphoantigens/*ex vivo* expanded γδ T cells, and allogeneic transplants containing γδ T cells. The year reported is the year of publication. The total number (*N*) of each disease treated and overall patients treated with each regimen are reported. Overall responses (OR) and complete responses (CR) from these reports are listed as numbers of patients responding over total patients with frequencies of response below. The OR was pooled partial and complete responses by RECIST (when applicable and reported) or by disease-free progression (when RECIST was not applicable or reported). References to the clinical trials are included in the far right column*.

Allogeneic γδ T cells have also been infused but were part of heterogeneous cell populations (Table 1). Patients with acute myelogenous leukemia (AML) and acute lymphoblastic leukemia (ALL) were treated with αβ T cell-depleted hematopoietic stem cell transplant (HSCT), which resulted in 100 objective responses (65%; *N* = 153) with 36 durable CRs (24%; *N* = 153) (66–69). These complete remissions could be directly correlated to the elevated persistence of donor-derived Vδ1 cells in the peripheral blood of the patients, suggesting that these cells were involved in long-term clearance of leukemia. Increases in peripheral Vδ1 cells have also been correlated with CMV re-activation in patients with leukemia following allogeneic HSCT (40, 70). Most recently, haploidentical PBMC were depleted of CD4^+^ and CD8^+^ cells using magnetic beads and were administered to patients with refractory hematological malignancies followed by Zol and IL-2 infusions (71). Three of the four patients treated experienced short-lived CRs (2, 5, and 8 months) and the other patient died of infection 6 weeks after treatment. Expansion of γδ T cells was observed the week after treatment suggesting that they may have directed the anti-tumor response. Currently, clinical trials of direct infusion of activated, homogenous populations of Vδ1 cells, or other non-Vγ9Vδ2 cells have yet to be undertaken but hold promise as future avenues of medical intervention.

## *Ex vivo* Propagation of Non-Vγ9Vδ2 γδ T Cells

Populations of γδ T cells outside of the Vγ9Vδ2 subset have been grown with immobilized TCRγδ agonists. Plate-bound recombinant MICA and IL-2 were used to sustain the proliferation of γδ T-cell cultures *ex vivo* from epithelial ovarian cancer and CRC tumor infiltrating lymphocytes (TILs) and resulted in high frequencies of Vδ1 cells (Figure 1B) (72). In addition, plate-bound pan-TCRγδ-specific antibody and IL-2 led to proliferation of both Vδ2 and Vδ1 cells (Vδ2 > > Vδ1) from peripheral blood derived from both healthy donors and patients with lung cancer or lymphoma (Figure 1C, top) (73, 74). Similarly, OKT3 has been used in combination with IL-2 and IL-4 to stimulate CD4/CD8-depleted T cells from healthy peripheral blood, which resulted in expansion of Vδ2 and Vδ1 cells (Vδ2 > Vδ1), albeit with reduced cell numbers compared to the TCRγδ monoclonal antibody (mAb)-stimulated cells (Figure 1C, middle) (75). A more complex cocktail of cytokines [IL-2, IL-12, and Interferon-γ (IFNγ)] has also been used with OKT3 and CD2-specific antibodies to expand γδ T cells, but the Vδ repertoires were not reported (Figure 1C, bottom) (76). Transition of these immobilized antigens and antibodies into clinical manufacture will streamline the application of these expansion strategies for γδ T cells and could be the source of clinical trials with non-Vγ9Vδ2 cells.

Highly polyclonal γδ T cells have been generated through co-culture of patient or healthy donor γδ T cells with irradiated artificial antigen presenting cells (aAPC), IL-2, and IL-21 (77–80). The aAPC (clone#4) are derived from the chronic myelogenous leukemia (CML) cell line K562 following genetic modification with T-cell co-stimulatory molecules (CD86 and CD137L), Fc receptors for antibody loading (introduced CD64 and endogenous CD32), antigens (CD19), and cytokines (a membrane-bound IL-15), and have been produced as a master cell bank (MCB) (81). This MCB is currently used in the production of αβ T cells for cancer treatments in clinical trials at MD Anderson (NCT01653717, NCT01619761, NCT00968760, and NCT01497184) (79, 82, 83). γ-irradiation of aAPC prior to co-culture with T cells subjects the aAPC to death (typically at or within 3 days) thereby reducing the risk for unintended transfer of this tumor cell line into recipients (83). Deniger et al. demonstrated that circulating γδ T cells, containing a polyclonal TCRγδ repertoire, could be isolated from healthy donor venipuncture or umbilical cord blood by “unlabeled/negative” magnetic bead selection and recursively stimulated with irradiated aAPC, IL-2, and IL-21 (Figure 1D, top). The aAPC-expanded γδ T cells proliferated to numbers sufficient for clinical use while maintaining the expression of most *TRDV* and *TRGV* alleles and demonstrating TCRδ surface expression of Vδ1 > Vδ1^neg^Vδ2^neg^ > Vδ2 (77). These polyclonal γδ T-cell cultures displayed broad tumor reactivity as they were able to lyse leukemia, ovarian cancer, pancreatic cancer, and colon cancer cells. Separation of the polyclonal cultures by TCRδ surface expression showed that each T-cell subset had anti-tumor reactivity and that a polyclonal γδ T-cell population led to the superior survival of mice with established ovarian cancer xenografts. Propagation of Vδ1^neg^Vδ2^neg^ cells had not been previously achieved and this was the first evidence of the functional activity of this γδ T-cell sub-population. In a similar study, Fisher et al. isolated polyclonal γδ T cells from PBMC of healthy donors or patients with neuroblastoma by first depleting monocytes followed by “positive/labeled” selection with anti-TCRγδ-hapten antibody and anti-hapten microbeads (Figure 1D, bottom) (79). This study made use of the Fc receptors on the aAPC surface to load anti-TCRγδ antibody where isolated γδ T cells were co-cultured with the antibody-loaded aAPC. These expanded γδ T cells expressed multiple *TRDV* and *TRGV* alleles with surface TCRδ expression of Vδ2 > Vδ1 > Vδ1^neg^Vδ2^neg^. Using this mode of expansion, Vδ1 and Vδ2 were mediators of antibody-independent (AIC) and antibody-dependent cellular cytotoxicity (ADCC), respectively, to neuroblastoma tumor cells (as predicted by whether or not they expressed Fc receptor CD16). aAPC-expanded polyclonal γδ T cells could be used for anti-tumor therapies because aAPC are currently available as a clinical reagent. However, human application of aAPC/mAb-expanded γδ T cells could depend on interest in the use of the current MCB of aAPC, generation of new MCB of aAPC at institutions where there are currently none, and production of γδ T cell agonistic antibodies in good manufacturing practice (GMP) conditions. Clinical testing of these cells could potentially lead to more widespread acceptance and use of γδ T cells as adoptive cellular therapies.

Given that the aAPC can sustain the proliferation of non-Vγ9Vδ2 cells to large quantities, there is opportunity for clinical translation, laboratory testing of subsets to elucidate their functions, and correlative studies. A limiting factor in studying γδ T cells has been the lack of TCRδ and TCRγ isotype-specific antibodies outside of specificity for TCRδ1, TCRδ2, TCRγ9, and TCRδ3 (where commercially available). Mice can now be immunized to generate mAb specific for desired TCRγδ isotypes where commercial and academic use of these detection antibodies can have tangible outcomes, including diagnostic and/or prognostic profiling of γδ T cells resident within tumors. γδ T-cell clones could be generated through co-culture of single γδ T cells with aAPC, and this can facilitate studies to determine Vδ/Vγ pairing, corresponding TCRγδ ligands, and pathogenic reactivity. The ligands on the K562-derived aAPC that TCRγδ binds are not currently known. Likely candidates include IPP and MICA/B for TCRδ2 and TCRδ1, respectively (22, 35). Elucidation of these interactions could assist attempts to tailor the design of the aAPC for total γδ T-cell expansion, propagation of a particular γδ T-cell lineage, or polarization toward a certain γδ T-cell phenotype (84). As an example, CD27^neg^ and CD27^+^ γδ T cells are associated with IL17 and IFNγ production, respectively (85–87), leading to the conclusion that expression of CD70, the CD27 ligand, on aAPC could potentially polarize these T cells toward a desired cytokine output. Thus, aAPC could be an excellent source for the study of fundamental γδ T-cell immunobiology and could yield answers not currently accessible because of limited starting cell numbers and ineffective polyclonal expansion protocols.

## Genetic Modification of γδ T Cells for Therapeutic Use

γδ T cells are also amenable to genetic modification allowing for the introduction of genes to improve their therapeutic function. For instance, re-directed specificity of T cells can also be accomplished through the introduction of recombinant TCRs with defined antigen specificity. The conventional thought is that transfer of TCRα/TCRβ genes into γδ T cells or transfer of TCRγ/TCRδ genes into αβ T cells would not cause mis-pairing with the TCRα/TCRβ and TCRγ/TCRδ heterodimers, thereby mitigating the risk of generating inappropriate pairings such as TCRα/TCRδ, TCRα/TCRγ, TCRβ/TCRγ, or TCRβ/TCRδ heterodimers with unknown specificity (88). This mis-pairing hypothesis was modeled in mice with the ovalbumin-specific αβ TCR OT-I, which resulted in re-directed specificity of murine γδ T cells toward ovalbumin peptide, but whether or not the TCRs were actually mis-paired was not reported (89). Vγ2Vδ2 cells have been expanded with 2M3B1PP and infected with γ-retrovirus to transduce TCRαβ chains with specificity toward MAGE-A4 peptide, but co-transduction with CD8 was required in order to transfer significant MHC Class-I-restricted recognition of MAGE-A4 peptide-pulsed tumor cells (90, 91). Similar studies have transferred αβ TCRs specific for CMV pp65 peptide or minor histocompatibility antigens into γδ T cells rendering them reactive to antigen-appropriate tumor cells (92). In contrast to the above reports of introducing αβ TCRs into γδ T cells, the Vγ9Vδ2 TCR has been transferred into αβ T cells and rendered both CD4^+^ and CD8^+^ T cells reactive to multiple tumor cell lines (93). Chemotherapy (temozolomide)-resistant γδ T cells have been generated by lentiviral transduction of (6)-alkylguanine DNA alkyltransferase into Vγ9Vδ2 cells expanded on Zol (94). Chimeric antigen receptors (CARs) can be introduced into T cells and re-direct the T cell toward a specific antigen. CARs are formed by fusing a single chain antibody to one or more T-cell intracellular signaling domains, e.g., CD3ζ, CD28, and/or CD137 (95). The antibody confers specificity through its variable regions toward a particular antigen, e.g., CD19, GD_2_, HER2, etc., and CAR binding to the antigen transmits intracellular T-cell signals for antigen-dependent proliferation, cytokine production, and cytolysis (96, 97). Following expansion on Zol, Vγ9Vδ2 cells were efficiently transduced to express CD19- and GD_2_-specific CARs with γ-retroviral vectors and displayed re-directed specificity toward CD19^+^ and GD_2_^+^ tumor targets, respectively (98). Zol and γ-retroviruses engineered to transduce CD19- and GD_2_-specific CARs are available for human application, but have not been combined in a clinical trial to date. Thus, subsets of γδ T cells are amenable to viral gene transfer to improve their therapeutic impact.

In contrast to γ-retroviruses and lentiviruses, which require cell division for efficient transduction, non-viral *Sleeping Beauty* (SB) transposition transfers genes into quiescent T cells and allows manipulation of cells that are difficult to culture *ex vivo* (99–102). SB transposase enzyme was originally derived from fish that were undergoing active transposition in their evolutionary maturation and was adapted for human application (103). In short, a DNA transposon with flanking inverted repeats and direct repeats is ligated into the human genome at TA dinucleotide repeats by the SB transposase enzyme (104). TA dinucleotide repeats are widely distributed in the human genome, yielding potential for random integration into the genome, and have been shown to be safe in regards to transgene insertion in pre-clinical studies (99, 101, 105). This is of particular importance in gene therapy as inappropriate integration at gene start sites or promoters, within exons, or even distal to genes within enhancers or repressors may cause cellular transformation. Lentiviruses and γ-retroviruses have higher efficiency in transgene delivery than SB, but these vectors are known to integrate near genes or within genes (97). Application of SB to human clinical-grade T cells has been reduced to practice as a two DNA plasmid system, where one plasmid contains the SB transposon with the transgene of interest, e.g., CAR, and the other plasmid encodes a hyperactive SB transposase (106). Electro-transfer of the DNA plasmids by nucleofection into circulating (quiescent) PBMC results in transient expression of SB transposase that then ligates the transposon into the genome using a “cut-and-paste” mechanism. As soon as the SB transposase mRNA is degraded translation of SB transposase protein is halted, thereby negating additional transposition events. T cells with stable CAR expression can be encouraged through the co-culture of T cells on irradiated aAPC that express antigen for the CAR (83). This process, originally developed for αβ T cells, has been adapted for expression of CAR in γδ T cells (78). Resting PBMCs were electroporated with CD19-specific CAR transposon and SB11 transposase plasmids and sorted the following day to deplete non-γδ T cells with magnetic beads from the transfected mixture. Isolated γδ T cells were recursively stimulated with CD19^+^ aAPC along with IL-2 and IL-21, which resulted in the outgrowth of CAR^+^ γδ T cells with a highly polyclonal TCRγδ repertoire. Endogenous leukemia reactivity by the aAPC-expanded γδ T cells was improved through expression of CD19-specific CAR rendering these T cells bi-specific through CAR and TCRγδ. SB transposon and transposase are available as clinical reagents; therefore, clinical trials can test the safety and efficacy of bi-specific CAR^+^ γδ T cells.

## Concluding Remarks

Given that γδ T cells are unlikely to cause graft-versus-host disease (GVHD) because their TCR ligands (IPP, MICA, etc.) are not MHC-restricted, γδ T cells (with or without genetic modification) could be generated from healthy donors in a third party manufacturing facility and given in the allogeneic setting as an “off-the-shelf” therapeutic. Additionally, a “universal” bank of polyclonal γδ T cells could be established that was known to have high anti-tumor immunity or contain a particular set frequency of Vδ1, Vδ2, and Vδ1^neg^Vδ2^neg^ populations to achieve superior efficacy (66). This could have specialized application in cases where T cells were difficult to manufacture, e.g., high tumor burden in blood or after extensive systemic (lymphodepleting) chemotherapy. Polyclonal γδ T cells could also be used as front-line therapy before addition of HSCT, CAR^+^ T cells, TILs, etc. in order to prime the tumor microenvironment for other adaptive immune cells with broader tumor specificity or to reveal neo-tumor antigens, including somatic non-synonymous mutations expressed only in the tumor (107–109). If immunity is restored in the recipients then the 3rd party γδ T-cell graft may be rejected, but there may still be a therapeutic window before this occurs. Both pro-tumor and anti-tumor effects of γδ T cells infiltrating the tumor microenvironment have been described (110, 111), and whether or not these cells could be useful for therapy could be delineated following expansion of γδ T cells from solid tumors on aAPC, which have been shown to expand TIL (αβ T cells) from metastatic melanoma (112). Tumor lysis by γδ T cells could lead to other resident cell types, e.g., NK cells, macrophages, αβ T cells, etc., to have renewed reactivity to the malignancy (113). Indeed, B-ALL cell lines coated with mAb were lysed by CD16^+^ Vγ9Vδ2 cells via ADCC, and subsequently the Vγ9Vδ2 had antigen presenting cell function to generate antigen-specific CD8^+^ αβ T cell responses to known B-ALL peptides, e.g., PAX5 (114, 115). Unknown is whether γδ T cells will be subjected to inhibition by regulatory T cells or other immunosuppressive forces. Some γδ T cells have been reported to have immunosuppressive function, and it would be of interest to identify these cells and eliminate them from the adoptive T-cell product prior to infusion (116). In summary, administration of graded doses of autologous and allogeneic, even 3rd party, γδ T cells in humans have tested and will continue to evaluate the ability of these lymphocytes to home and recycle effector function in the tumor microenvironment. Given the development of aminobisphosphonates, synthetic phosphoantigens, immobilized antigens, antibodies, and designer clinical-grade aAPC, it now appears practical to sculpt and expand γδ T cells to achieve a therapeutic effect.

## Author Contributions

Drew C. Deniger, Judy S. Moyes, and Laurence J. N. Cooper wrote the manuscript.

## Conflict of Interest Statement

Dr. Cooper founded and owns InCellerate, Inc. He has patents with Sangamo BioSciences with artificial nucleases. He consults with Targazyme, Inc. (formerly American Stem cells, Inc.), GE Healthcare, Ferring Pharmaceuticals, Inc., and Bristol-Myers Squibb. He receives honoraria from Miltenyi Biotec. Other authors declare no other competing financial interests.

## Abbreviations

2M3B1PP, 2-methyl-3-butenyl-1-pyrophosphate; AML, acute myeloid leukemia; BrHPP, bromohydrin pyrophosphate; CLL, chronic lymphocytic leukemia; CRC, colorectal cancer; EOC, epithelial ovarian cancer; FCL, follicle center lymphoma; GI-cancer, cancers from the gastrointestinal tract; HIV, human immunodeficiency virus; HRPC, hormone-refractory prostate cancer; IC, immunocytoma; MM, multiple myeloma; MZL, mantle zone lymphoma; N/D, not determined; NHL, T-cell non-Hodgkin lymphoma; NSCLC, non-small-cell lung cancer; RCC, renal cell carcinoma; TBI, total body irradiation; T-SPL, secondary plasma cell leukemia; Zol, zoledronic acid.
